# Ketamine as a therapeutic agent in major depressive disorder and posttraumatic stress disorder: Potential medicinal and deleterious effects

**DOI:** 10.1002/ibra.12094

**Published:** 2023-02-20

**Authors:** Bhuvi Sachdeva, Punya Sachdeva, Shampa Ghosh, Faizan Ahmad, Jitendra Kumar Sinha

**Affiliations:** ^1^ Department of Physics and Astrophysics, Bhagini Nivedita College University of Delhi Delhi India; ^2^ GloNeuro Academy Noida Uttar Pradesh India; ^3^ ICMR—National Institute of Nutrition Tarnaka Hyderabad India; ^4^ Department of Medical Elementology and Toxicology Jamia Hamdard Delhi India

**Keywords:** anesthetic agent, antidepressant, neuroplasticity, neurotoxicity, therapeutics

## Abstract

Major depressive disorder (MDD) and posttraumatic stress disorder (PTSD) are the most common causes of emotional distress that impair an individual's quality of life. MDD is a chronic mental illness that affects 300 million people across the world. Clinical manifestations of MDD include fatigue, loss of interest in routine tasks, psychomotor agitation, impaired ability to focus, suicidal ideation, hypersomnolence, altered psychosocial functioning, and appetite loss. Individuals with depression also demonstrate a reduced behavioral response while experiencing pleasure, a symptom known as anhedonia. Like MDD, PTSD is a prevalent and debilitating psychiatric disorder resulting from a traumatic incident such as sexual assault, war, severe accident, or natural disaster. Symptoms such as recalling event phases, hypervigilance, irritability, and anhedonia are common in PTSD. Both MDD and PTSD pose enormous socioeconomic burdens across the globe. The search for effective treatment with minimal side effects is still ongoing. Ketamine is known for its anesthetic and analgesic properties. Psychedelic and psychotropic effects of ketamine have been found on the nervous system, which highlights its toxicity. In this article, the effectiveness of ketamine as a potential therapeutic for PTSD and MDD along with its mechanisms of action, clinical trials, and possible side effects have been discussed.

## Graphical abstract

Ketamine is known for its anesthetic and analgesic properties. Its primary mechanism involves blocking the *N*‐methyl d‐aspartate (NMDA) receptor and indirectly activating α‐amino‐3‐hydroxy‐5‐methyl‐4‐isoxazolepropionic acid (AMPA) receptor. In this article, the mechanism of ketamine action, clinical trials, and effectiveness of ketamine as a potential therapeutic approach for PTSD and MDD are discussed. Ketamine helps in repairing cortical damage in patients with MDD and PTSD. It stimulates BDNF signaling and neuroplasticity, and has an antidepressant effect.

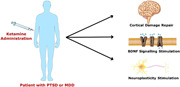

## 1 INTRODUCTION

**Figure 1:**
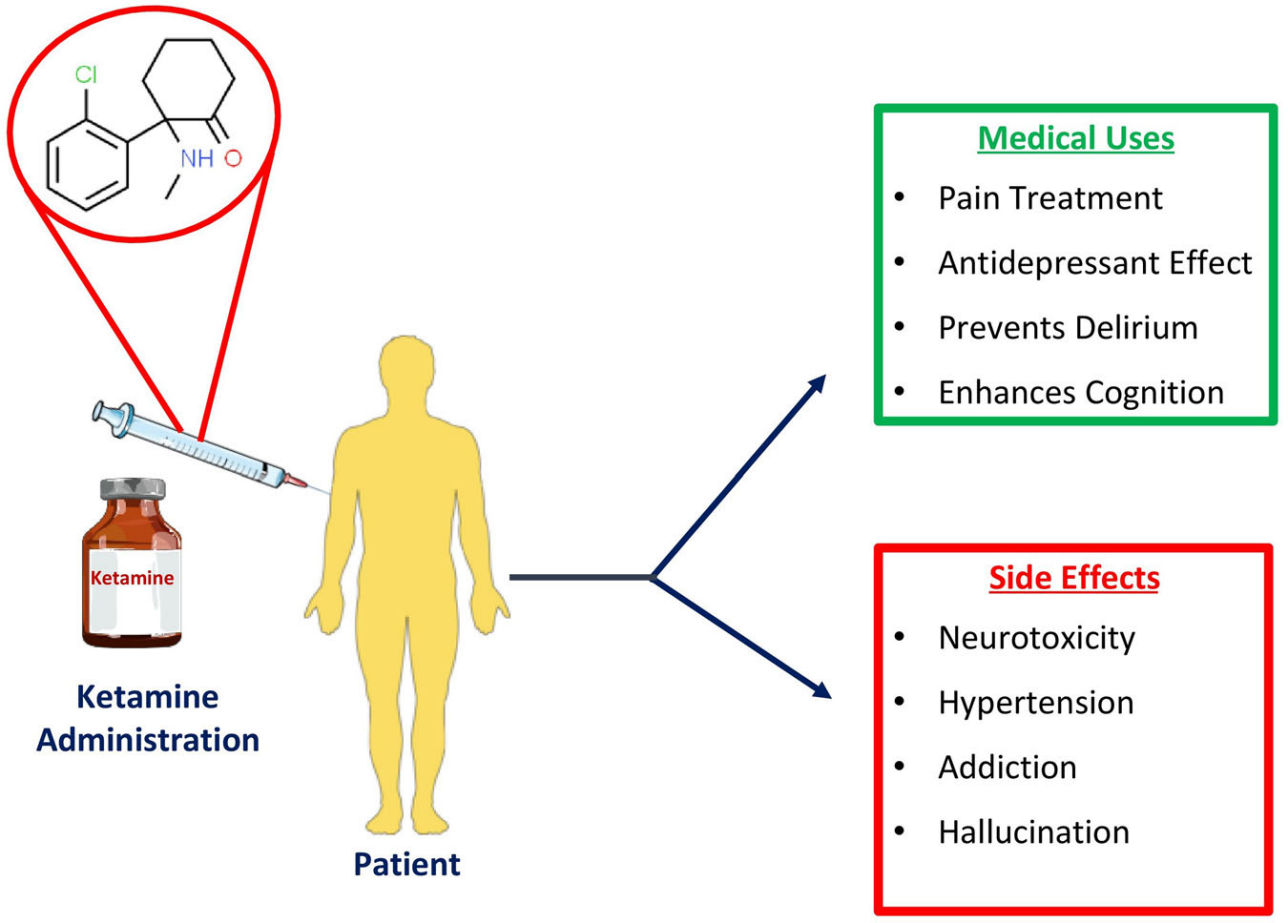
Chemical structure, medical uses, and possible side effects associated with the administration of ketamine. [Color figure can be viewed at wileyonlinelibrary.com]

Ketamine, an anesthetic agent, was first developed by Calvin Stevens at Parke Davis in the 1960s as an analog of phencyclidine (CI‐581).^1^ In 1964, it was accepted for human trials,^1^ and 20 prisoners in Jackson prison in Michigan, USA, were the first to get ketamine administered.^2^ Ketalar (ketamine hydrochloride) emerged as the first Food and Drug Administration (FDA)‐approved ketamine sample for individual utilization.^3^ Ketamine (an arylcyclohexylamine) has one chiral center and two enantiomers: (R)‐ketamine (or ketamine) and (S)‐ketamine (or esketamine).^4^ The medical uses, side effects, and chemical structure of ketamine are shown in Figure 1. It acts by blocking the *N*‐methyl‐d‐aspartate receptor (NMDA).^5^ (S)‐ketamine shows a higher affinity for NMDAR than (R)‐ketamine. Therefore, it was manufactured as an antidepressant.^6^ In addition, S‐ketamine has higher analgesic and anesthetic effects than R‐ketamine and causes fewer psychotic and other adverse effects.^7^ Although both (R)‐ketamine and (S)‐ketamine have been described to exert antidepressant results independently,^8^ R‐ketamine has greater antidepressant effects than S‐ketamine, without ketamine‐related side effects.^9^ Moreover, preclinical studies on an animal model with depression showed that (R)‐ketamine has the potential to exert antidepressant effects for a more extended period, and (R)‐ketamine has fewer deleterious side effects than (R, S)‐ketamine and (S)‐ketamine.^10^ While (R)‐ketamine and (S)‐ketamine have different affinities for the NMDAR, (R)‐ketamine shows more substantial and more enduring antidepressant‐like effects in animal models of depression. Importantly, in rodents, monkeys, and humans, (R)‐ketamine induces fewer negative side effects than (R, S)‐ketamine or (S)‐ketamine. A recent pilot trial showed that (R)‐ketamine produced both quick‐acting and long‐lasting antidepressant benefits in patients with depression who were resistant to therapy.^11^ The most usual metabolic conversion is *N*‐demethylation into nor‐ketamine. The molecule fulfills Lipinski's rules, that is, the measures used to evaluate the oral action of the drug. In the United States, ketamine is a Schedule III and a widespread street drug.^11^ There is now just one pharmacological class of drug that has been licensed for the treatment of the symptoms of posttraumatic stress disorder (PTSD), which is generally a challenging mental health condition to treat. Although the selective serotonin reuptake inhibitors (SSRIs) have a low risk of side effects, unfortunately, it is challenging to achieve a complete remission of PTSD symptoms. Atypical antipsychotics, hypnotics, mood stabilizers, and anxiolytics are examples of off‐label augmentation possibilities, but the use of these drugs for the treatment of PTSD is not efficient. Given that it reduces NMDA receptor activation, ketamine is probably used more frequently for anxiety disorders. Although it is acknowledged that the controlled drug ketamine is also constrained by the offset of effects over 1–2 weeks, which limits its usage in treatment‐resistant MDD.^12^ Moreover, it is generally one of the most abused drugs at raves and among spiritual seekers as it has the potential to elicit out‐of‐body experiences (OBEs).^6^ OBEs are hallucinatory visual experiences in which the physical body is perceived as existing in a different visual environment. These events have apparently been linked to a wide variety of psychiatric conditions, neurological issues, pharmaceutical drugs, and altered psychological states.^13^ OBEs have been associated with a number of brain lesions, especially in the parietal and temporal regions, mental diseases, intense emotional states like a near‐death experience, substance abuse, migraines, and epilepsy, but relatively few have been recorded in dissociative identity disorder.^14^ Ketamine has numerous names like cat valium, purple, vitamin K, cat tranquilizer, special la coke, super acid, and jet special K. One of the most common ways to administer ketamine is through nasal ingestion.^3^ The patient generally enters a dissociative state after ketamine is administered at sedation doses. This state is different from the state evoked by other sedatives, wherein the patient is not unarousable, as observed with medicines like propofol and etomidate, but rather, their sensory inputs are disconnected from their conscious state. Patients can speak and have rare movements on intake of this medication; despite remaining unaware of their surroundings, patients will frequently have eye movements, including several patterns of nystagmus.^15^ A case series published by Weiner et al. recorded anxiety, palpitations, chest pain, agitation, and tachycardia as some of the clinical presentations in ketamine abusers.^16^ This review of literature aims to understand how ketamine can modulate the symptoms of patients suffering from PTSD and depression. Moreover, the toxic effects of ketamine have also been discussed further in the article.

## 2 OVERVIEW ON DEPRESSION

Depression is a long‐term mental condition that affects 300 million people globally.^17^ Some of the symptoms include lack of energy, loss of interest in everyday tasks, psychomotor agitation, diminished capacity to focus, suicidal ideation, hypersomnolence, altered psychosocial functions, and loss of appetite globally.^18^, ^19^ The risk of developing major depression is influenced by both genetics and the environment. It has been suggested that epigenetic mechanisms increases in the risk of depression following exposure to traumatic life events and offer a mechanistic framework for integrating hereditary and environmental components. The term “epigenetics” refers to biological mechanisms that regulate gene expression and translation but do not entail modifications to the DNA sequence. These processes include histone modifications, microRNAs, and DNA methylation (DNAm).^20^ Depression is multifactorial and it is still not clear as how it precipitates differentially in different individuals. When individuals have a severe medical condition, depression may occur. Others may experience depression when their lives change, such as when they lose a loved one. Some individuals have a history of depression in their families. People who suffers from depression usually experience an overwhelming melancholy and loneliness for no specific reason.^17^ In 2008, the World Health Organization classified depression as the third most common disease, and projected that disease will rank the first by 2030.^19^ The brain of individuals with depression has a different structural and functional appearance. According to data from magnetic resonance imaging (MRI), patients with depression have gray matter loss, a reduction in the total brain volume, altered hippocampal volume, amygdala atrophy, striatal atrophy, and reduced serotonin transporters in the amygdala. Therefore, it has been confirmed in numerous studies that depression as a neurodegenerative disorder resulting in atrophy of the brain regions.^21^ Neurodegenerative disorders develop due to loss of neurons, resulting in a decline in cognitive functioning of the brain.^22^, ^23^, ^24^, ^25^, ^26^, ^27^, ^28^ Several studies have identified the hippocampus as the key brain region linked to depression, and hippocampal plasticity occurs in patients with depression.^29^ Interestingly, nutritional deficiencies have been observed to initiate and exacerbate depression and anxiety.^30^, ^31^ Furthermore, research has found a link between inflammation and depression. Elevated concentrations of inflammatory markers such as interleukin‐6 (IL‐6), tumor necrosis factor‐alpha, C‐reactive protein, and the soluble IL‐2 receptor have been identified in depressive patients, indicating that their inflammatory response is disrupted. The pathophysiology of depression is also influenced by nitrosative and oxidative stress. In patients with depression, elevated cortisol levels, stress axis dysregulation, and reduction in brain‐derived neurotrophic factor (BDNF),^32^, ^33^ increase in insulin‐like growth factor‐1, and fibroblast growth factor‐1 have all been reported.^34^

### 2.1 Neurochemistry of depression: The monoamine hypothesis

The monoamine hypothesis of major depressive disorder states that a reduction in certain neurotransmitters, such as dopamine, serotonin, and norepinephrine (NE), in the brain induces clinical depression. Also, this theory has been proven in the case of antidepressant intake. Antidepressants play a major role in the increase of these three neurotransmitters, which eventually decreases depressive symptoms.^17^ Many studies have found that depression arises from dysfunction in serotonergic, dopaminergic, and noradrenergic systems. However, some recent studies show that monoamine deficiency cannot lead to depression and that increasing the levels of neurotransmitters cannot help to cope with symptoms of depression.^17^ The “serotonin hypothesis” of clinical depression has been around for more than 50 years. The most basic claim of the hypothesis is that the pathophysiology of depression is caused by decreased serotonin pathway activation. This hypothesis was based on the depressogenic effects of amine‐depleting substances like reserpine as well as the effects of antidepressant medications like monoamine oxidase inhibitors and tricyclic antidepressants, which were later found in animal experimental studies to potentiate the effects of serotonin and other monoamines at the synapse. These substances were discovered by clinical spontaneity.^35^ The neurotransmitter dopamine is responsible for motivation, reward prediction, and responsiveness to daily activities; dysfunction in the dopamine system leads to anhedonia, which is one of the most common symptoms of depression.^36^ There is ample evidence that NE is involved in depression, and current research on neural circuits and symptoms has highlighted the unique function of NE in this condition. NE controls executive functioning, which governs cognition, motivation, and intellect—three factors crucial to social interactions. One of the most significant factors influencing the quality of life of people with depression may be social dysfunction.^37^

## 3 DEEPER INSIGHTS INTO PTSD

Many individuals encounter potentially traumatic situations in their lives that lead to posttraumatic stress symptoms for a short period; this is a common reaction. In PTSD, the neurotransmitters that are mainly involved are serotonin, GABA, and glutamate.^38^ Most people can cope with a stressful situation with the help of others, but about 10% of these individuals develop PTSD. Whether an individual develops PTSD is determined by the time of exposure, the age at which the stressful event occurred, and the type of traumatic experience.^39^ PTSD is a mental condition that develops after traumatic experiences like sexual assault, military warfare, a major vehicle accident, kidnapping, natural disasters, and so forth.^40^, ^41^ It was first studied when symptoms were seen in people in Civil War I and people who got exposed to military trauma. In the early stages of PTSD, within days of being exposed to trauma, clinical signs appear.^42^, ^43^ Recall event phases, hypervigilance, hyperarousal, dysphoria, or anhedonia are some of the most typical symptoms.^44^ PTSD is more common among police officers, emergency medical personnel, military soldiers, and firefighters, according to the Diagnostic and Statistical Manual of Mental Disorders, Fifth Edition (DSM‐5). It is linked to poor academic performance, increased incidences of depression, suicide attempts, and substance addiction, and other psychological problems.^45^ Many risk factors for PTSD have been empirically classified. These can be divided into three broad categories: preexposure, peritraumatic, and postexposure factors. Preexposure factors include neurobiological factors such as endowment, genes, and epigenetic modifications, as well as environmental factors such as personal psychiatric history, stressful life, poor living conditions, trauma exposure, and lower education, along with behavioral factors such as higher emotional reactivity and impaired executive function.^45^ The nature and severity of the trauma are peritraumatic elements. Social assistance and “secondary” stressors such as job loss resulting from the tragedy are examples of postexposure factors.^46^ Anatomical and functional anomalies in the frontolimbic circuitry, which carries out emotion regulation and threat processing tasks, were detected during neuroimaging evaluations of persons with PTSD. In addition, the ventromedial prefrontal cortex and the dorsal anterior cingulate cortex have reduced gray matter volume.^44^ A cross‐sectional study of PTSD patients found a decrease in hippocampal volume and increased amygdala reactivity. In the 1980s and the 1990s, psychological debriefing was widely utilized to prevent PTSD symptoms.^47^ This therapy facilitated rapid emotional processing of traumatic experiences soon after exposure to an incident,^48^ but some research has shown that debriefing negatively impacted the recovery process.^49^ Cognitive behavioral therapy (CBT) has also been useful in managing some of the symptoms of PTSD.^50^ Hydrocortisone is effective in the treatment of PTSD in people who have never been treated for psychiatric illnesses.^49^ For the treatment of PTSD, hydrocortisone appears to be a promising and affordable drug. The need for additional, thorough research in this area is highlighted by the very few studies and the poor methodological quality of these studies.^51^

## 4 KETAMINE: AN EMERGING ANTIDEPRESSANT

The therapeutic options for depression mainly rely on the usage of antidepressants, mostly monoaminergic agents, such as tricyclic antidepressants, monoamine oxidase inhibitors, selective SSRIs, and dual serotonin–norepinephrine reuptake inhibitors. The effectiveness of these agents depends on the hypoactivity of the monoamine neurotransmitter system (predominantly NE, dopamine, and serotonin), leading to the pathophysiology of depression.^52^ Unfortunately, present pharmacologic therapeutic interventions are ineffective for depression since their therapeutic benefits take weeks to manifest, and only one‐third of patients with depression receive proper treatment. Individuals suffering from treatment‐resistant depression commonly struggle without effective pharmacological treatment, resulting in suicidal ideation.^53^ Clinical evidence has shown that an intravenous infusion of low‐dose ketamine (0.5 mg/kg) just once has a rapid antidepressant effect that continues to be effective for at least 1 week.^54^ In addition, studies have also shown ketamine to be an effective antidepressant in patients with treatment‐resistant depression who also have suicidal ideation.^53^

## 5 EFFECT OF KETAMINE ON MDD PATIENTS

**Figure 2:**
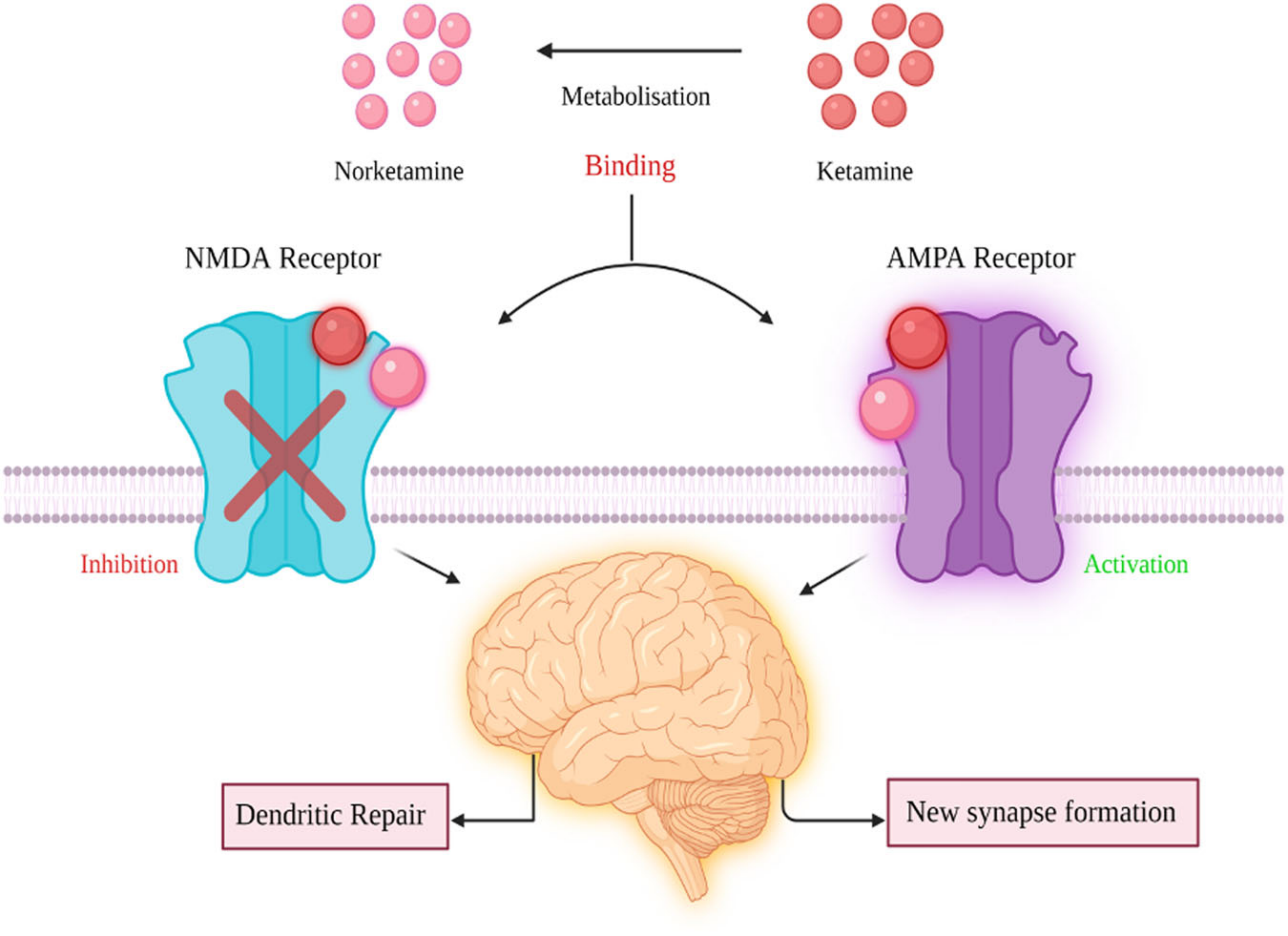
Effect of ketamine metabolites acting on *N*‐methyl d‐aspartate (NMDA) and α‐amino‐3‐hydroxy‐5‐methyl‐4‐isoxazolepropionic acid (AMPA) receptors. The binding of ketamine or nor‐ketamine will inhibit the activity of the NMDA receptor. In contrast, if ketamine binds to the AMPA receptor, it will be activated and will initiate downstream signaling activities; both the inhibition of the NMDA receptor and activation of the AMPA receptor will lead to the formation of new synapses and repair of neuronal dendrites, enabling reversal of the adverse effects of depression or stress associated with posttraumatic stress disorder in patients. [Color figure can be viewed at wileyonlinelibrary.com]

Major depression is defined by increased activation or sensitivity to noxious stimuli in limbic emotion processing and subcortical areas and insufficient activation or inadequate functioning in prefrontal cortical regions involved in emotion regulation and cognitive control processing.^54^ Ketamine has been recognized as a rapid‐acting antidepressant therapy in many studies. It targets the protein complex NMDAR, which is present on the surface of neurons, as mentioned previously. According to studies, inhibition of NMDAR proteins with ketamine is effective in managing the depressive‐like behavior more quickly and than traditional antidepressant medications.^55^ It inhibits GluN2B, which is one of the subunits of NMDAR protein. Experiments on humans and rats have revealed that pharmacological agents with the ability to inhibit this subunit (GluN2B) will have antidepressant‐like characteristics.^56^ Miller, Yang, and colleagues created a rodent line (called 2BCtx) that lacks the GluN2B subunit in excitatory neurons to study the effects of ketamine on NMDAR proteins. They carried out two tests to assess the baseline levels of depressive‐like symptoms in 2BCtx mice: the forced‐swim test and the tail‐suspension test. In these experiments, a rodent with depressive‐like symptoms spends less time moving than a wild‐type rodent, indicating behavioral despair, one of the hallmarks of depression in humans. The 2BCtx rodents spent more time moving than the wild‐type rodents throughout both tests, implying that they show fewer depressive‐like symptoms.^57^ In patients with depression, new research links specific variations in a network of subcortical, prefrontal, and limbic brain areas.^58^ According to one theory, ketamine enhances α‐amino‐3‐hydroxy‐5‐methyl‐4‐isoxazolepropionic acid (AMPA) and NMDA receptor function and density by directly blocking NMDA receptors and indirectly increasing AMPAR function and density as shown in (Figure 2), which activates downstream synaptogenic signaling pathways, restoring synaptic connectivity and strength in the prefrontal cortex and the hippocampus.^59^ Amino acid, neuropeptide, monoamine, and neuroendocrine transmitter systems influence behavioral changes by influencing the connection of neurons inside our brain.^58^ Ketamine acts by bringing these mood‐regulating mechanisms back into balance. According to positron emission tomography, ketamine increases neuronal activation in the prefrontal cortex (PFC).^60^ It was also discovered to activate the mammalian target of rapamycin pathway quickly, leading to an increase in synaptic signaling proteins and improved function and a number of new spine synapses in mouse PFCs.^61^ These findings support ideas relating the antidepressant effects of NMDA receptor antagonists to an increase in neuroplasticity and neurotrophic‐related factors, as well as neurotrophic theories of depression in general.^62^ Although it is clear that ketamine exerts antidepressant effects by altering emotion regulation and processing systems in the brain, the exact nature of these changes is still unknown.^60^

## 6 EFFECT OF KETAMINE ON PTSD PATIENTS

Paroxetine and sertraline are the only two permitted drugs approved by the FDA for treating symptoms of PTSD.^63^ A survey was distributed to several PTSD researchers, asking them to suggest five plausible new pharmacological treatment targets for PTSD to better understand pharmacotherapies that should be investigated to treat the condition. As a result, glucocorticoid receptor agonists, NMDA receptor antagonists, opioid receptor antagonists, cannabinoid receptor agonists, and non‐SSRI antidepressants were selected as the first five pharmacological treatment targets for PTSD.^64^ As a result, among experimental medications, rapid‐acting glutamatergic agents and ketamine are considered the best choices. The effectiveness of ketamine over midazolam in chronic PTSD patients was investigated in a clinical study.^41^ The use of ketamine resulted in a significant reduction in the severity of PTSD symptoms. This was the first study to report an active reduction in the severity of symptoms in patients with chronic PTSD after receiving ketamine infusion.^41^ The activation of the NMDA receptor has been linked to the creation of uncontrollable intrusive memories, and high NMDA receptor function is associated with the development of PTSD.^65^ A study conducted by Zhang and colleagues examined the development of PTSD in mice after they were exposed to a context‐specific fear, based on the fact that intrusive thoughts are linked to activation of the NMDA receptor. After administering an electric shock to their feet, these rodents were subjected to a time‐dependent sensitization technique (TDS). The rats were restrained for 2 h before being placed in a cylinder‐shaped pipe full of water and forced to swim for 20 min; following a 15‐min break, they were administered diethyl ether. Zhang and colleagues were able to recreate the TDS after a 1‐week recovery period. During this time, a ketamine dose was administered to the rodents based on their weight. The first‐line therapy was sertraline, an SSRI, which was administered to the positive controls. By counting the number of crossing and rearing with lines, Zhang and colleagues ruled out the possibility that ketamine had an influence on the rodents' mobility and activity. Rearing is a method of measuring exploratory and active behaviors in mazes and open fields. No significant change in rearings or line crossings was observed in the experimental and control groups of rodents. This confirmed that neither ketamine nor traumatizing stimuli were involved in causing anxiety or psychosis when used with TDS exposure. Starting from the first day after the TDS procedure, ketamine or sertraline was administered regularly. The hippocampus of animals with post‐TDS had considerably lower levels of BDNF in post‐mortem examinations.^66^ Chronic ketamine treatment reduced the impact of the stressful incident and increased BDNF levels. This is significant because the hippocampus, in particular, is linked to memory and learning, and so is the hypothalamus–pituitary–adrenal axis (HPA). Antagonizing NMDA receptors have been shown to hinder fear training consolidation in the hippocampus.^67^ According to a study, individuals in emergency care or the Intensive Care Unit who have experienced stressful situations are much more prone to developing PTSD. Sedative/anesthetic drugs can interfere with memory formation mechanisms, increasing or decreasing the memory of a traumatic experience and, as a result, increasing or decreasing the likelihood of developing PTSD.^68^ Several investigations have shown that a breakdown of synaptic connectivity induces the symptoms of PTSD. Stress linked to PTSD may decrease synaptic connectivity, which is normally mediated by glutamate.^69^ Due to the critical function of glutamate synapses in these brain circuits, ketamine intervention may repair synaptic connections in these circuits, correcting the impact of stress.^70^ Table 1 presents a summary of different clinical trials conducted using ketamine in both PTSD and MDD patients.

**Table 1:** Different clinical trials of ketamine conducted on PTSD and MDD patients.

S. no.	Name of the disorder	Result	References
1.	PTSD & MDD	When ketamine was administered at a dose of 0.2–2 mg/kg to patients, improvements in MDD symptoms were observed, and this is still in Phase II trials for PTSD	[71]
2.	PTSD	An unknown dose of ketamine was administered to a patient, with no effect on PTSD symptoms	[72]
3.	Chronic PTSD	0.5 mg/kg of ketamine was administered to patients reduce PTSD symptoms rapidly	[73]
4.	Chronic PTSD	10 mg of ketamine reduces physical aggression and decreases emotion dysregulation in patients	[74]
5.	PTSD	An unknown dose of ketamine was administered to the patient, which did not show any effect on the symptoms	[75]
6.	Chronic PTSD	0.5 mg/kg of ketamine was administered to the patient; it reduced PTSD symptoms	[76]
7.	PTSD	When the unknown dose of ketamine was administered to the patient, it reduced symptoms of PTSD	[77]

Abbreviations: MDD, major depressive disorder; PTSD, posttraumatic stress disorder.

## 7 KETAMINE AND NEUROTOXICITY

Ketamine exerts psychedelic and psychotropic effects on the central nervous system. Psychedelic side effects depend on the amount of dose infused during the treatment of chronic pain. The perception of reality is affected, causing panic attacks, increased awareness of sound and space, auditory hallucinations, visual hallucinations, paranoid ideas, and inability to control thoughts. Moreover, some patients experience intense euphoria, dizziness, vertigo, nausea, blurred vision, nystagmus, vivid dreams, memory deficits, dysphasia, and impaired motor function. An assessment of cognitive and memory functioning during a short duration of treatment with ketamine revealed impairments in working memory and declines in data encoding into episodic memory. Moreover, in contrast to different amnestic drugs, it induces semantic memory impairment.^77^ Psychedelic effects of ketamine show psychological dependence long‐term abuse.^78^ Recurrent ketamine abusers show more damage to the brain by causing neurotoxicity. Studies in the developing rodent brain reported that NMDAR antagonists induce neurodegeneration through apoptosis, and enhancement of excitatory neuronal activity in the brain causes neuronal injury.^79^ Liao et al. observed the toxicity of ketamine in an adult brain in two studies. They reported that brain volume in ketamine abusers decreases, that is, degeneration in white matter was observed in the temporoparietal cortex and reduced gray and white matter volume was observed in the bilateral frontal cortex. Studies suggest that patients with a history of drug abuse should not be treated with ketamine.^65^ It exerts indirect stimulatory and direct inotropic effects on the cardiovascular system. Activation of the sympathetic nervous system induces stimulation and is associated with inhibition of NE reuptake, vagal nerve inhibition, and catecholamine release at peripheral nerves. High or low ketamine doses induce myocardial depression, which is characterized by increased myocardial oxygen consumption and cardiac output, tachycardia, and pulmonary hypertension.^80^ Some reports also show that ketamine treatment leads to increased liver enzyme profile^81^; continuous low‐dose and high‐dose ketamine infusions led to a 10% elevated liver enzyme profile and it took 3 months for this to return to normal. Although the mechanism by which ketamine induces injury in the liver is not entirely understood, some plausible factors include an increase in lipid peroxidation along with the production of free radicals, a decrease in hepatic oxygen, and allergic hepatitis.^82^ These findings demonstrate the adverse effects of ketamine when used in uncontrolled settings. While on ketamine treatment, patients should be continuously monitored and treatment must be discontinued if any side effects are observed. Additional side effects of ketamine have been observed in recreational ketamine users, including urinary abnormalities, chronic schizotypal behavior, and memory problems.^83^ An increase in caspase‐3‐ and Fluoro‐Jade C‐positive neuronal cells was observed in the frontal cortex of rats administered six injections of 20 mg/kg ketamine. The typical nuclear condensation and fragmentation observed under electron microscopy indicated heightened apoptotic features. Other parts of the brain also showed increased cell death, which can lead to neurodegeneration.^84^

## 8 ANATOMICAL VARIATIONS IN KETAMINE USERS

Neuroanatomical alterations were investigated among ketamine and non‐ketamine users.^83^ Patients who had used ketamine for a more extended period were shown to have less gray matter volume in the right middle frontal gyrus and the left superior frontal gyrus.^83^ In an additional MRI study, reduced cortical thickness was observed in various regions of the right frontal cortex in chronic ketamine users. There was no mention of how ketamine was administered. To determine how the effects of ketamine on the brain increase over time, a subsequent structural MRI study examined scans of 21 individuals. In chronic ketamine users who had been addicted to the drug for a long time, between 0.5 and 12 years,^85^ alterations in both the gray and white matter in the cerebellum, internal capsule, diencephalon, and basal forebrain were observed. Prolonged use of ketamine was linked to substantial cortical atrophy in the occipital, parietal, frontal cortex, and parahippocampal gyrus.

Interestingly, patients who had been addicted to ketamine for 3 years or less than 3 years showed less atrophy than those addicted to ketamine for more than 2 years.^85^ On 124 chronic ketamine users, a structural MRI record showed reduced volume of lower gray matter in the left hippocampus (IH), the right medial prefrontal cortex, the left globus pallidus, the right orbitofrontal cortex, and the right nucleus accumbens.^86^ Contrary to other studies, this study found more gray matter volume in the left caudate nucleus in ketamine users. Liang et al. (2020) observed structural changes in ketamine users and discovered that ketamine users have larger white matter and caudate nucleus volume. Ketamine users who frequently used stimulants had even larger white matter volume, suggesting that ketamine and stimulants have an addictive effect.^86^

## 9 FUTURE CHALLENGES

The Ketamine and metabolites of ketamine are essential in developing novel pharmacotherapies that do not have ketamine's adverse effects, such as psychotomimetic side effects, sensory perception alteration, and possibilities for misuse.^87^ Besides recent experimental depression models, ketamine metabolites are also being investigated in animal studies of pain, inflammation, depression, and suicidal behavior.^87^ Extensive clinical use of racemic ketamine, (S)‐ketamine, (R)‐ketamine, and significant metabolites presents an enormous opportunity to develop novel medicines for unmet medical needs and to better understand pharmacology–phenotype connections. Given the current state of knowledge regarding the safety of racemic and enantio‐pure ketamine when administered promptly, clinical evaluation of these medicines is feasible. The researchers are now focussing on the developments in alternate routes of administration, dosing techniques, and drug combination research. Additionally, when (S) ketamine was synthesized from (R, S) ketamine to provide (S) ketamine as an anesthetic medication in many countries, (R) ketamine was initially refused. If (R) ketamine can exert immediate and prolonged antidepressant effects in patients with MDD without causing adverse effects, it may represent as therapeutic drug for several mental diseases. Finally, elucidating novel molecular and cellular targets involved in the rapid and prolonged antidepressant effects of ketamine and its enantiomers will aid in the development of novel antidepressants without ketamine's adverse side effects.^82^ Furthermore, the breadth of potential evidence linked to ketamine's various pharmacological targets provides a unique opportunity to develop new antidepressants without the harmful side effects of ketamine. Given these considerations, it is evident that a basic understanding of ketamine and ketamine metabolite pharmacology opens up a wealth of opportunities in both basic and translational research.^15^ Recent observations on various physiological experiments show that maintaining sleep quality could be beneficial in reducing the overall pain sensation and depression‐like symptoms.^88^, ^89^ Dose–response studies, especially trials that use more acceptable modes of administration or different doses, are therefore strongly advocated.

Alternative methods for boosting ketamine's antidepressant effects should also be investigated. The effects of ketamine can also be seen to be differentially precipitated due to various health conditions including diabetes, obesity, cardiovascular conditions, cancer, and other.^90^, ^91^, ^92^, ^93^ Given these proposed mechanisms of action, other drugs that boost prefrontal plasticity are expected to enhance an antidepressant effects.^94^ FDA clearance of S‐ketamine for treatment‐resistant MDD is a defining moment in the field of psychiatry. If ketamine or ketamine‐like therapies for the most severe forms of depression and other mental illnesses are reintroduced, these medications could significantly improve the quality of life for millions of currently untreated individuals. While the efficacy of ketamine in treating further neuropsychiatric disorders has not been shown, preliminary studies are intriguing. As noted previously, preliminary evidence suggests that ketamine may be useful in the treatment of obsessive‐compulsive disorder, PTSD, and MDD.^69^ While these findings are encouraging for the many people who suffer from severe depression, they must be weighed against the reported and possibly unknown adverse effects of long‐term ketamine treatment. For instance, some case reports have asserted that ketamine produced fixation, despite laboratory findings indicating that a single ketamine infusion did not affect mood in bipolar individuals.^95^ During the last two decades, preclinical and clinical ketamine research has laid the groundwork for ongoing research into novel methods of regulating and treating this severe condition.^96^

## 10 CONCLUSION

Ketamine has been used as an anesthetic agent since the 1960s. Ketamine, as observed in animal research, clinical trials, and case reports, significantly reduces PTSD‐related symptoms in a short duration of time. Similarly, it may have a rapid, substantial antidepressant effect in patients suffering from depression. Additionally, long‐term safety and efficacy concerns should be investigated further, and adverse reactions should be evaluated routinely. However, ketamine exerts psychedelic and psychotropic effects on the central nervous system. Psychedelic side effects are dosage‐dependent and are also dependent on the dose administered during chronic pain therapy. The perspective of reality is altered, resulting in panic attacks, enhanced awareness of sound and place, auditory and visual illusions, paranoid thinking, and an inability to manage thoughts. Additional research is needed to determine the optimal dose and manner of administration for ketamine's antidepressant efficacy and to elucidate its modes of action; with future investigations, ketamine may emerge as a promising option for treating PTSD and depression in patients who have failed to respond to more traditional treatments.

## AUTHOR CONTRIBUTIONS

Bhuvi Sachdeva, Punya Sachdeva, Shampa Ghosh, and Jitendra Kumar Sinha contributed to the conceptual framework, data collection, data curation, writing, and making figures in the manuscript. All the listed authors have read and approved the final manuscript.

## CONFLICT OF INTEREST STATEMENT

The authors declare no conflict of interest.

## ETHICS STATEMENT

Not applicable.

## Data Availability

Data generated during the study are reported and avaliable from corresponding author for reasonable request.
